# BARD1 Autoantibody Blood Test for Early Detection of Ovarian Cancer

**DOI:** 10.3390/genes12070969

**Published:** 2021-06-25

**Authors:** Maxim Pilyugin, Magdalena Ratajska, Maciej Stukan, Nicole Concin, Robert Zeillinger, Irmgard Irminger-Finger

**Affiliations:** 1 Department of Gynaecology and Obstetrics, University of Geneva, 1211 Geneva, Switzerland; Maxim.Pilyugin@unige.ch; 2 Department of Gynecology and Obstetrics, Medical University of Gdansk, 80210 Gdansk, Poland; mratajska@gumed.edu.pl; 3 Department of Pathology, Otago Medical School, University of Otago, Dunedin 9016, New Zealand; 4 Gdynia Oncology Center, Department of Gynecologic Oncology, 81519 Gdynia, Poland; maciej.stukan@gmail.com; 5 Department of Gynaecology and Obstetrics, Medical University of Innsbruck, 6020 Innsbruck, Austria; Nicole.Concin@i-med.ac.at; 6 Molecular Oncology Group, Department of Obstetrics and Gynecology, Comprehensive Cancer Center, Medical University of Vienna, 1090 Vienna, Austria; robert.zeillinger@meduniwien.ac.at; 7 BARD1 Life Sciences Limited, Melbourne 3000, Australia

**Keywords:** *BRCA1*, *BARD1*, breast cancer, ovarian cancer, autoantibodies, blood test, early detection

## Abstract

Background: Ovarian cancer (OC) is the most lethal gynaecological cancer. It is often diagnosed at an advanced stage with poor chances for successful treatment. An accurate blood test for the early detection of OC could reduce the mortality of this disease. Methods: Autoantibody reactivity to 20 epitopes of BARD1 and concentration of cancer antigen 125 (CA125) were assessed in 480 serum samples of OC patients and healthy controls. Autoantibody reactivity and CA125 were also tested for 261 plasma samples of OC with or without mutations in *BRCA1/2*, *BARD1*, or other predisposing genes, and healthy controls. Lasso statistic regression was applied to measurements to develop an algorithm for discrimination between OC and controls. Findings and interpretation: Measurement of autoantibody binding to a number of BARD1 epitopes combined with CA125 could distinguish OC from healthy controls with high accuracy. This BARD1-CA125 test was more accurate than measurements of BARD1 autoantibody or CA125 alone for all OC stages and menopausal status. A BARD1-CA125-based test is expected to work equally well for average-risk women and high-risk women with hereditary breast and ovarian cancer syndrome (HBOC). Although these results are promising, further data on well-characterised clinical samples shall be used to confirm the potential of the BARD1-CA125 test for ovarian cancer screening.

## 1. Introduction

Ovarian cancer (OC) is a major cause of cancer-related deaths for women. A woman’s risk of developing OC during her lifetime is about 1 in 78. Her lifetime chance of dying from OC is about 1 in 108 in the US [1,2,3,4]. 

OC is only the eighth most frequent cancer diagnosed in women but has the lowest survival rate. This is mostly due to its detection at a late stage, while early detection and treatment increases the treatment’s success and survival. High-risk group women with hereditary breast and ovarian cancer (HBOC) or women with a family history of breast and/or ovarian cancer have 10 times increased risk of developing OC. The most frequently mutated genes in HBOC women and women with sporadic breast or OC are *BRCA1* and *BRCA2* [5]. 

Lifetime cancer risks in *BRCA* mutation carriers are 60–80% for breast cancer and 20–40% for OC. Besides *BRCA* gene mutations, other susceptibility genes can be mutated or their expression modified, including genes of the Fanconi anemia (FA) cluster (*FANCD2, FANCA,* and *FANCC*), mismatch repair (MMR) cluster (*MLH1*, *MSH2*, *PMS1*, *PMS2,* and *MSH6*), DNA repair cluster (*ATM*, *ATR,* and *CHK1/2*), and tumour suppressor cluster (*TP53*, *SKT11,* and *PTEN*) in HBOC women [6]. For both, the high-risk HBOC and average-risk women, early detection would permit better treatment with a higher probability for success.

Morphologically, OC is recognised as a highly heterogeneous group of tumours, with epithelial ovarian tumours accounting for about 90% of cases and being further grouped into five main histological subgroups of ovarian cancer including subtypes serous, endometroid, mucinous, clear cell, and transitional cell tumours (Brenner tumours) [7]. Currently, treatment depends mostly on the grade and stage of the tumour [8]. However, progress in genetic testing might lead to the new classification and improved risk assessment of OC [9]. Besides these developments, it is clear that early detection can save lives by providing successful treatment prior to metastasis. This indicates that simple, non-invasive methods such as liquid biopsy for early detection are needed.

Current biomarkers for OC include CA125 either alone or combined as multivariate tests with an algorithm that enhances the predictive strength of these biomarkers. However, CA125 is not sensitive for early-stage OC [1]. Existing diagnostic tests such as OVA1, ROMA, and OVERA include a number of biomarkers in addition to CA125, in particular human epididymal protein 4 (HE4) and an algorithm including menopausal status [10] (reviewed in Meta-analysis [11]. 

The International Ovarian Tumour Analysis group (IOTA) has a multifaceted algorithm that has been systematically evaluated in Europe to high acclaim [12]. There have also been attempts to simplify the IOTA algorithm [13,14]. Other morphology-based indices have been proposed and validated in the US and elsewhere [15,16,17]. Moreover, much similar to longitudinal CA125 (ROCA), serial transvaginal ultrasound (TVUS) offers improved diagnostic results over a single evaluation [18,19]. 

Although the accuracy of OC tests is improving, none of the existing tests is recommended for screening but rather as a diagnostic aid for women identified with a pelvic mass. The *BARD1* gene is a low-risk predisposition gene for breast/ovarian cancer in its own right [20,21,22]. The BARD1 protein acts with BRCA1 as a ubiquitin ligase in repair pathways [23,24]. In ovarian, breast, and other cancers, mRNA and protein isoforms of BARD1, generated by exon skipping, are overexpressed and correlated with tumour progression, while the full-length gene expression was abrogated [25,26,27]. 

Although *BARD1* has been categorised as a low penetrance predisposition gene for OC, its contribution to OC might be epigenetic and posttranscriptional. Expression of differentially spliced BARD1 isoforms has been described in all morphological subtypes of OC [25]. Furthermore, BARD1 mRNA and protein isoforms have been described as oncogenic, and their expression correlated with poor patient survival. These isoforms therefore could be useful biomarkers for early detection and could trigger an autoimmune response. A truncated protein isoform of BARD1 was identified to provide immune protection from colon cancer in a murine cancer model [28]. 

It has been postulated that due to altered BARD1 molecules, cancer patients generate an immune response and autoimmune antibodies against various epitopes of BARD1, which could be detected as biomarkers of cancer [29,30]. Indeed, autoimmune antibodies against BARD1 were found in OC patients when using an in vitro generated isoform of BARD1 as antigen. Autoimmune antibodies in sera of cancer patients were reactive to epitopes on BARD1 isoforms and could be exploited to develop an inverse ELISA assay for early detection of lung cancer [31]. 

Here, we report on a blood test specifically developed for early detection of OC using autoantibodies to immunogenic sites on BARD1 and its isoforms.

## 2. Materials and Methods

### 2.1. Patients Samples

Serum samples from 280 patients with OC and 200 healthy controls (age-matched women without symptoms) were obtained from three different sources: University Hospitals of Geneva (Switzerland), Medical University of Vienna (Austria), and BioServe Biotechnologies, Ltd. (Beltsville, MD, USA). Information on age and diagnosis of ovarian cancer type and stage (According to International Federation of Gynaecology and Obstetrics, FIGO) is shown in Table 1.

Plasma samples were obtained from 127 OC patients with or without mutations in breast/ovarian cancer predisposition genes (Table S1) and 134 age-matched healthy women as controls (Table 2) from Pomeranian Hospitals, Gdynia Oncology Centre, Gdynia (Poland). Informed consent for the scientific use of biological material was obtained from all patients and healthy female blood donors in accordance with the requirements of the local ethics committees of the involved institutions. Blood from women undergoing genetic testing is most often prepared as plasma. Hence, we used plasma samples for comparison of the BARD1 autoantibody test performance in samples from women with and without mutations in breast/ovarian cancer predisposition genes.

All serum and plasma samples were taken from first-time ovarian cancer patients at the time of diagnosis.

### 2.2. Antigen Selection

A set of 20 peptides of 10 to 20 amino acids in length chosen from predicted antigenic sites of the protein sequences of full-length BARD1 or BARD1 isoforms, published previously [31], was used in this study.

### 2.3. ELISA Assays

For BARD1 ELISA assays, peptides were custom spotted onto wells of 96-well plates by the plate manufacturer (Meso Scale Discovery (MSD), Rockville, MD, USA) at approximately 0.05 μg per spot. Antigens were incubated with serum or plasma samples from ovarian cancer patients and control samples from healthy women, and MSD electrochemiluminescence assays were performed according to the manufacturer’s specifications. Plates were incubated with PBS-5% Blocker A solution for 1 h at room temperature. Blocking solution was discarded, and 25 μL of serum samples diluted at 1:200 in PBS-1% Blocker A were added and incubated for 2 h at RT. After washing with PBS-0.05% Tween, 25 μL of anti-human SULFO-TAG detection antibody (# R32AJ-5, Meso Scale Discovery), diluted to 1.2 μg/mL in PBS 1% Blocker A, was added and incubated for 1 h at RT. After three PBS-0.05% Tween washes, 150 μL 2× MSD Read Buffer T was added to the wells, and plate reading was immediately performed on the MSD Sector Imager 2400. To minimise experimental variability, cancer and control samples were randomly distributed on each plate. The samples were measured in duplicates. Each plate contained wells probed with known anti-BARD1 antibodies against epitopes that were tested previously in various studies [26,27,32,33]. 

For CA125 assays, CA125 levels were measured using MSD Human Cancer Antigen 125 Assay 96-Well MULTI-ARRAY (MSD Catalogue Number K151IWC-2), according to the instructions provided by the CA125 kit supplier. To minimise experimental variability, cancer and control samples were randomly distributed on each plate.

### 2.4. Statistical Analysis

#### 2.4.1. LASSO Modelling

The lasso logistic regression modelling method was used for the primary data analysis, as described [31,34]. The glmnet R package was used for fitting lasso logistic regression [35,36]. 

#### 2.4.2. Lasso ROC Analysis

**OptimalCutpoints** [37,38] and pROC [39] packages were used for generating and analysing ROC curves including computation of AUC sensitivity, specificity, and corresponding confidence intervals.

#### 2.4.3. Cross-Validation

For cross-validation, the Monte-Carlo method was used to randomly divide the 480 serum sample data set 200 times in 2/3 training (187 cancer, 133 controls) and 1/3 validation (93 cancer, 67 controls) sets. The model fitted to each training set was then applied to the corresponding test set (validation). The average AUC, sensitivity, and specificity were computed for 200 training and test subsets. The same 200 subsets were used for all computations.

## 3. Results

### 3.1. A BARD1 Autoimmune Antibody Test to Distinguish OC and Controls

To investigate the value of BARD1 autoantibodies in the detection of OC, we used 20 BARD1 epitopes for antibody capture in inverse ELISA tests and measured antibody binding in 480 serum samples from 280 OC patients and 200 healthy control women.

**Figure 1:**
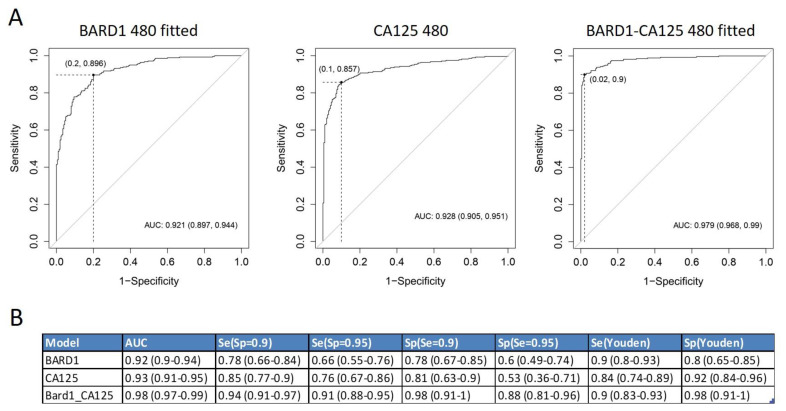
Roc curves of BARD1 480-sample-fitted model, compared to CA125 and BARD1-CA125 480-sample-fitted model: (**A**) ROC curves for BARD1 480-sample-fitted model, CA125, and BARD1-CA125 480-sample-fitted model are shown with optimal cut-offs (Youden); (**B**) the specificity and sensitivity values for BARD1 480-sample-fitted (BARD1), CA125, and BARD1-CA125 are shown for different cut-offs. Se (sensitivity), Sp (specificity), AUC (area under the ROC (receiver operator characteristic) curve).

The results of antibody binding showed that more than one peptide was needed to optimally discriminate between cancer and controls. Applying Lasso logistic regression, an algorithm was generated which included and scored a combination of peptides that distinguished OC from controls with high accuracy (Figure 1). The 480-sample-fitted model reached an area under the ROC curve (AUC) of 0.921. This corresponds to a sensitivity of 0.895 and a specificity 0.8 at the Youden cut-off, a sensitivity of 0.78 at a specificity of 0.9, or a sensitivity of 0.66 at a specificity of 0.95. The model consisted of 19 peptides.

### 3.2. BARD1 Autoimmune Antibody Test Compared and Combined with CA125

We compared the accuracy of the BARD1 480 sample-fitted model with CA125, the most established biomarker for OC monitoring (Figure 1). The CA125 ROC curve for the 480 samples reached an AUC of 0.928. This corresponds to a sensitivity of 0.84 and specificity of 0.9, at the Youden cut-off, or a sensitivity of 0.76 at a specificity of 0.95.

We next combined the BARD1 480 antibody values and the CA125 protein concentration values in a Lasso model (Figure 1). This BARD1-CA125 480-sample-fitted model included CA125 and 13 peptides.

The BARD1-CA125 480-sample-fitted model reached an AUC of 0.979, which corresponds to a sensitivity of 0.9 and specificity of 0.98 at the Youden cut-off. The BARD1-CA125 480 test reached a sensitivity of 0.91 at a specificity of 0.95. Hence, the BARD1-CA125 480 model reached better accuracy than either BARD1 480 fitted or CA125 alone.

**Figure 2:**
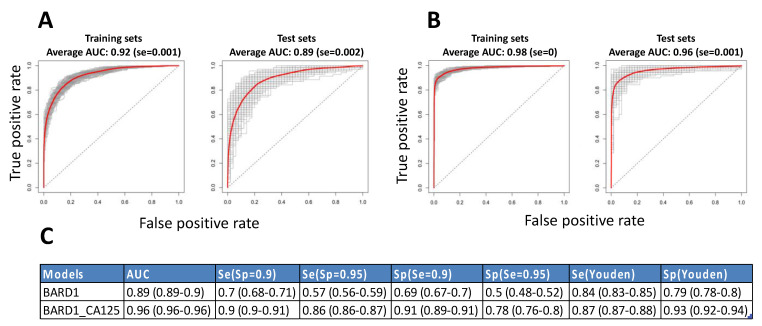
Cross-validations for BARD1 models based on 20 peptides without or with CA125 included. For cross-validation, antibody binding data for 480 samples were randomly and proportionally split 200 times in 3⁄4 for training sets (187 cancers, 133 controls) and 1⁄4 validation sets (93 cancers, 67 controls). Lasso modelling was performed on antibody binding data of each of the 200 training sets and applied on complementary validation sets: (**A**) the respective ROC curves obtained for models of 200 training and 200 (left) validation sets (right) are presented and average ROC curves are marked red; (**B**) the respective ROC curves obtained for 200 training (left) and validation sets (right) for BARD1-CA125 models are presented and average ROC curves are marked red; (**C**) the specificity and sensitivity values for average ROC curves of test sets are shown for different cut-offs for BARD1 and BARD1-CA125 models. The 95% confidence intervals are shown in brackets.

To statistically validate the BARD1 480-sample-fitted and the BARD1-CA125 480-sample-fitted models we performed statistical cross-validation by applying Monte-Carlo simulation for splitting the 480-sample set into two—a training set containing 320 samples (two-thirds of the total) and a validation set containing 160 samples (one-third of the total) repeatedly 200 times. Each time the training set model was applied to the corresponding independent validation set. The ROC curves for the training and validation or test sets showed an average ROC curve AUC that was similar to the BARD1 480-sample-fitted ROC curve of 0.92 with different ROC curves ranging from 0.94 to 0.88 (Figure 2A). The AUC of the average ROC curve of the test sets was 0.89, but the AUCs for the different ROC curves ranged from 0.92 to 0.7.

Similarly, we performed cross-validation by building BARD1-CA125 fitted models on 200 training sets and applying them on 200 validation sets (Figure 2B). The average ROC curve AUC was 0.98 for training sets and 0.96 for test sets. The sensitivity based on the average ROC curve of the validation sets was 0.86 at the specificity cut-off of 0.95 (Figure 2C). The range of the AUCs of the different ROC curves of the test sets was much smaller for the BARD1-CA125 models than for the BARD1 peptides models.

### 3.3. BARD1 Autoimmune Antibody Test and CA125 for Different OC Stages

**Figure 3:**
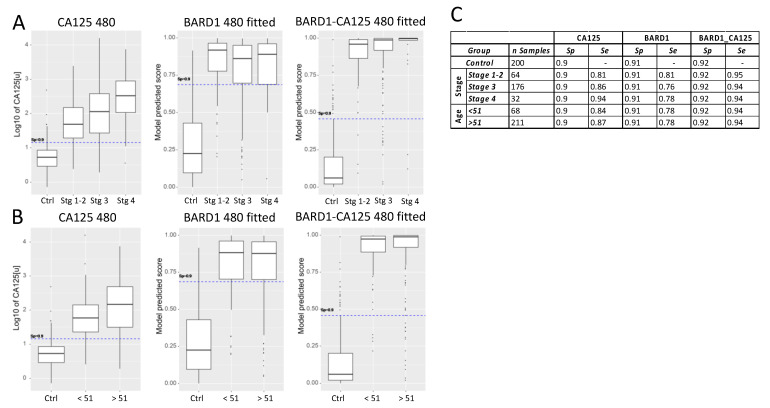
Sensitivity analysis of CA125, BARD1 480-sample-fitted model, and BARD1-CA125 480-sample-fitted model for different ovarian cancer stages: (**A**) the CA125 values (log10 scale) or the scores predicted by BARD1 480-sample-fitted and BARD1-CA125 480-sample-fitted models are shown for controls and cancer stages. The cut-off was fixed at specificity 0.9 (dashed line); (**B**) comparison of sensitivity of CA125, BARD1 480-sample-fitted model, and BARD1-CA125 480-sample-fitted model by age group of <51 and >51 (51 and more); (**C**) the specificity and sensitivity values for groups of age are shown for specificity =0.9 cut-off.

We then investigated the accuracy of the BARD1 480-sample-fitted model and the BARD1-CA125 480-sample-fitted model, in comparison with CA125 alone, for different ovarian cancer stages (Figure 3).

CA125 sensitivity for different stages of ovarian cancer, at a specificity of 0.9, was 0.81 for combined stages I and II, 0.86 for stage III, and 0.94 for stage IV (Figure 3). The BARD1 480-sample-fitted model showed an AUC of 0.81 for stages I and II, 0.76 for stage III, and 0.78 for stage IV, which was similar to that of CA125 for early stages but less than CA125 for late stages. However, the combined BARD1-CA125 480-sample-fitted model showed an increased accuracy over both the CA125 and the BARD1 480-sample-fitted models at all stages, with a sensitivity of 0.95 at stages I and II and 0.94 at more advanced stages at a specificity cut-off of 0.92 (Figure 3A,C). The CA125 accuracy increased with tumour stages, while the BARD1-CA125 480 test showed equally high accuracy at all stages.

In order to address the question of how post-menopausal status influences the accuracy of either BARD1 480-sample-fitted, CA125, or BARD1-CA125 480-sample-fitted prediction models, we applied the models to two age groups: those of less than 51 and those 51 years of age and more (Figure 3B). We used 51 as a surrogate cut-off as we did not collect menopausal status for the patients. CA125 was slightly more accurate for the older, presumed post-menopausal age group, at a specificity cut-off of 0.9. The BARD1 480-sample-fitted model had equal sensitivity for both groups, and the BARD1-CA125 480-sample-fitted model showed equally high accuracy for both groups (Figure 3B,C).

The required accuracy of a test depends on the prevalence of the disease. We, therefore, calculated the positive predictive value (PPV) and negative predictive value (NPV) for the BARD1 480 and BARD1-CA125 480 average cross-validation test groups, as compared to CA125 (Table 3).

Using data from the National Cancer Institute (2016) for the average population and prevalence for *BRCA* mutation carriers [40], we determined the PPV and NPV for the general population and *BRCA* mutation carriers, as well as for women of different age groups (Table 3).

### 3.4. BARD1 Autoantibody Test for Women with HBOC

**Figure 4:**
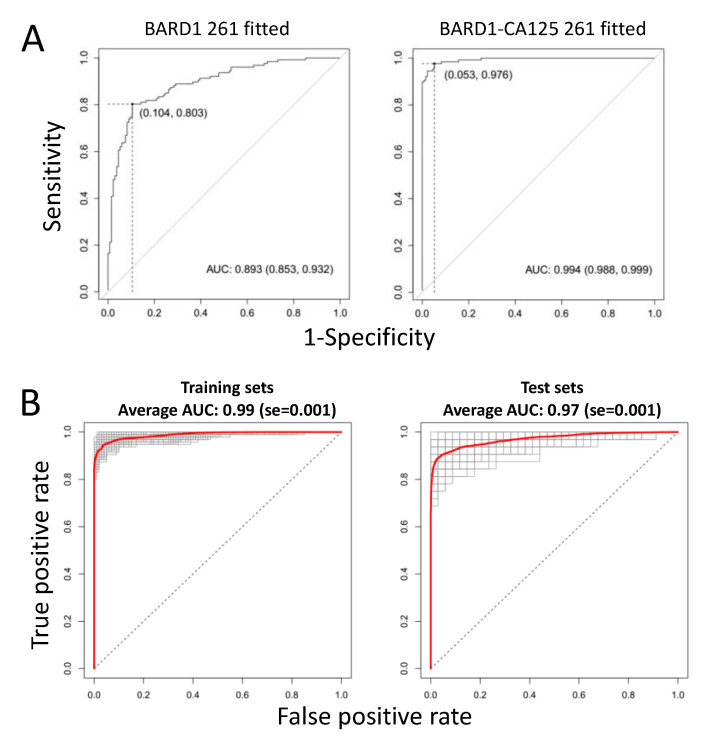
ROC curves for BARD1 261 -sample-fitted and BARD1-CA125 261-sample-fitted models: (**A**) ROC curves for BARD1 261-sample-fitted and BARD1-CA125 fitted models with optimal cut-offs (Youden) are shown; (**B**) the respective ROC curves of BARD1-CA125 models obtained for 200 training and 200 (left) validation sets (right), performed as for Figure 2, are presented, and average ROC curves are marked red.

To determine the performance of the BARD1 OC test in women with mutations in *BRCA1*, *BRCA2*, or *BARD1*, or other predisposition genes, a pilot study was performed using the 20-peptide BARD1 panel for ELISA tests for measuring BARD1 autoimmune antibodies and CA125 in 261 plasma samples including samples from ovarian cancer patients with or without mutations in *BRCA1/2* or other predisposition genes and healthy controls. Algorithms were generated based on the BARD1 261 plasma samples autoantibody data or autoantibody data CA125 values. The resulting models reached similar or slightly higher AUCs (Figure 4A) than the BARD1 480-sample-fitted models obtained for serum samples (Figure 1). The BARD1 261-sample-fitted model included 13 peptides, 12 of those were present also in the BARD1 480 model. This shows that an independent sample set and independent modelling resulted in similar peptide selection and level discrimination of OC and controls.

The predictors for the BARD1-CA125 261 model included 12 BARD1 peptides. Cross-validation by using repeatedly (200 times) three-quarters of the plasma samples as test sets and the complementary one-quarter of the samples as validation sets showed that the average AUC for test sets was 0.97 for the BARD1-CA125 261 models (Figure 4B), which was similar to that obtained for the BARD1-CA125 480-sample-fitted models.

**Figure 5:**
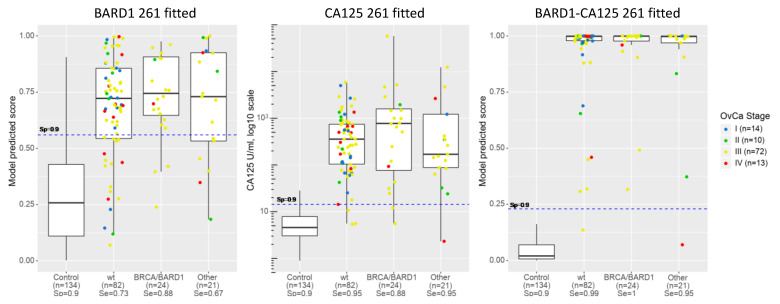
BARD1 261-sample-fitted model, BARD1-CA125 261-sample-fitted model, and CA125 applied to ovarian cancers with or without mutations. Comparison of model prediction score of BARD1 261-sample-fitted model, CA125, and BARD1-CA125 261-sample-fitted model applied to healthy controls (Control), ovarian cancers without known mutation (wt), with BRCA1/2 and BARD1 mutations (BRCA/BARD1), and with other than BRCA1/2 or BARD1 mutations (Other). Prediction scores or concentration (CA125) are presented as dots for each sample and as boxplots. Sensitivity (Se) is shown for controls and each group of ovarian cancers under each label. Distribution of cancer stage (I to IV) is indicated by colour code for each group.

The efficacy of the models was determined for prediction of OC in subgroups of the 261 cohort, patients with or without *BRCA1/2* or other mutations, or healthy women (Figure 5). CA125 showed higher sensitivity (shown at the cut-off of specificity 0.9) for the no-mutation (wt) OC group and the “other” OC group than the *BRCA1/2* or *BARD1* mutation group. CA125 also showed higher sensitivity than the BARD1 261 fitted model. The *BRCA1/2* or *BARD1* mutation group showed equally high sensitivity with both CA125 and BARD1 261-sample-fitted models. However, the BARD1 261-sample-fitted model showed the highest sensitivity for the *BRCA1/2* or *BARD1* mutation group. Importantly, the BARD1-CA125 261-sample-fitted model reached a sensitivity of 0.99 for the no-mutation (wt) and “other” groups (other than *BRCA1/2* or *BARD1* group of OC) and a sensitivity of 1 for the ovarian cancers with *BRCA1/2* or *BARD1* mutations.

These results suggest that a model trained on OC samples from women with and without *BRCA1/2* mutations could be a screening tool for the detection of OC in HBOC women.

## 4. Discussion

The data presented above demonstrate that the combination of autoantibodies to several immunogenic BARD1 peptides, together with assessment of CA125 levels, can potentially improve on CA125 alone as a test for the detection of early-stage OC and for detection of OC independent of hormonal status (Figure 3). Current OC tests include measured CA125 concentrations and patient age and/or hormonal status in the algorithm. Including age and/or hormonal status in the algorithm of the BARD1-CA125 OC test is likely to further improve its accuracy.

Due to the low prevalence of OC, an efficient screening method for detecting early-stage OC would require a sensitivity greater than 75% with a specificity of at least 99.6% and a positive predictive value (PPV) of at least 10% in order to be accepted [41,42]. The PPV for the BARD1 test was not increased over the PPV for CA125 alone. Whilst the PPV for the combined BARD1-CA125 model was higher, it is too low for a screening test for the general population; the predicted PPV for *BRCA1/2* mutation carriers indicated that the BARD1-CA125 test could be acceptable for screening of women with known *BRCA1/2* mutations or HBOC.

Women with HBOC syndrome, due to mutations in *BRCA1/2* or other breast/ovarian cancer predisposition genes, are more likely to develop breast and ovarian cancer and are advised to have annual mammography tests for early detection of breast cancer. However, no reliable monitoring test exists for early detection of OC for HBOC women.

The BARD1-CA125 tests proved to be specifically sensitive for the detection of ovarian cancer in women with *BRCA1/2* or *BARD1* mutations. CA125 showed higher sensitivity than the BARD1 261-sample-fitted model for OC with or without *BRCA1/2* or *BARD1* mutations, presumably this reflects that those sub-groups contained more late-stage ovarian cancers, which are better detected by CA125 than early-stage cancers. It is expected that in a mixed population, screened for early stages of OC with and without predisposing mutations, the BARD1 261-sample-fitted model would prevail over CA125. The combined BARD1-CA125 OC test, however, as shown with the BARD1 261-CA125 model, would show high sensitivity and specificity (AUC 0.97) for OC regardless of disease stage, menopausal status, or mutations in breast/ovarian cancer predisposition genes.

These data can be compared to other existing biomarkers and combinations thereof, in particular the diagnostic value of the ovarian malignancy algorithm (ROMA). A meta-analysis evaluated retrospectively the ROMA index for 5954 cases [43]. The pooled estimates for the ROMA index were a sensitivity of 0.90, specificity of 0.91, and area under the ROC curve of 0.96.

Our data suggest that a test based on combining assessment of BARD1 autoantibodies, together with the measurement of CA125 levels, would be a viable assay for regular screening of women with known HBOC. The relatively high accuracy of the BARD1-CA125 model could also be useful for testing symptomatic women. As most women with ovarian cysts or similar benign diseases must undergo inspective laparoscopy, which is an invasive surgery, it would be ideal to have a non-invasive test that could determine with high accuracy the malignant or benign nature of the disease. This could reduce the number of diagnostic surgical interventions and improve the selection of patients who need to be referred to a gynaecologic oncologist. In this case, the BARD1-CA125 test, if positive, would be a decision aid to perform diagnostic surgery to detect a potentially malignant tumour.

Measuring autoantibody binding to a number of epitopes of a protein rather than to the whole BARD1 protein or its isoforms is novel. Modelling of the antibody binding data is an approach that accounts for the variability in immune reactivity to epitopes in patient (or control) populations and for the fact that antibody titre or binding strength does not correlate with tumour progression. The caveat of this approach is that it needs sufficiently large cohorts to generate a robust algorithm.

The robustness of statistical models was assessed by splitting samples into training and validation sets, which is a standard statistical method to assess the performance of a test (algorithm) on an independent sample set. This procedure predicted acceptable sensitivity and specificity for the BARD1-CA125 test for a given patient population (Figure 2). The shortfall of this method, compared to one independent sample set, is that (i) the unique optimal algorithm determined for the whole sample set cannot be tested and (ii) the influence of samples from different origins is diluted because of the random mixture of samples in training and test sets regardless of origin.

Such an influence of origin or ethnicity cannot be overlooked, and stringent protocols for blood taking, as well as preparation and storage of samples, will have to be applied to minimise this effect.

We performed the modelling for a BARD1 OC test for two independent cohorts, resulting in similarly accurate discrimination of OC and controls. A total of 741 samples were collected at four different medical centres, supporting the conclusion that the performance of the BARD1 OC or BARD1-CA125 OC tests is likely to be reproducible in different settings. It will be important to determine how accurately the BARD1-CA125 OC test can discriminate ovarian cancer from benign ovarian lesions. Whether other diseases can influence the accuracy of the BARD1-CA125 OC test remains to be elucidated. Future work including testing of more samples from different origins, with a diverse composition of age, hormonal status, ovarian cancer stage and grade, and genetic background of breast/ovarian cancer predisposition genes will ultimately confirm the predicted accuracy. To progress the development of this BARD1-CA125 OC test towards a future possible application, a transfer of the test onto a platform used in routine laboratories would be preferable.

## Figures and Tables

**Table 1:** Serum samples of ovarian cancer and study control populations.

Samples	Property	Ovarian Cancer				Control			
		UHI	HUG	BioServe	Total	UHI	HUG	BioServe	Total
Number		198	38	44	280	100	50	50	200
Age	Range	22–86	37–90	38–75	22–90	NA	50–76	22–80	22–80
	Median	63	62	56	62	NA	53	60	54
Stage	I	42	7	1	50	NA	NA	NA	NA
	II	13	1	1	15	NA	NA	NA	NA
	III	108	26	41	175	NA	NA	NA	NA
	IV	28	3	1	32	NA	NA	NA	NA
	Unknown	7	1	0	8	NA	NA	NA	NA

**Table 2:** Plasma samples of ovarian cancer and study control populations.

Samples	Property	Ovarian Cancer	Controls
Number		127	134
Age	Range	22–86	31–68
	Median	52	46
Mutation status	wt	82	NA
	Fam	45	NA
	BRCA1/2	24	NA
	Others *	21	NA

Mutation status: wild type (wt); familial breast/ovarian cancers (Fam). * ATM, BLM, Check2, MRE11A, NBM, PALB2.

**Table 3:** PPV and NPV for reference groups.

Model	Cutoff		General		BRCAm		<50		50–64		>65	
			PPV	NPV	PPV	NPV	PPV	NPV	PPV	NPV	PPV	NPV
CA125	Sp 0.95	Se0.76	0.16%	99.997%	8.94%	99.837%	0.06%	99.999%	0.33%	99.994%	0.57%	99.991%
	Sp 0.99	Se0.62	0.66%	99.996%	28.60%	99.753%	0.24%	99.999%	1.33%	99.992%	2.27%	99.986%
	Se0.95	Sp 0.53	0.02%	99.999%	1.29%	99.939%	0.01%	100.000%	0.04%	99.998%	0.08%	99.996%
	Se0.99	Sp 0.18	0.01%	99.999%	0.77%	99.964%	0.00%	100.000%	0.03%	99.999%	0.05%	99.998%
	Youden		0.11%	99.998%	6.35%	99.888%	0.04%	99.999%	0.23%	99.996%	0.39%	99.993%
BARD1 CV validation set average	Sp 0.95	Se0.57	0.12%	99.995%	6.86%	99.708%	0.04%	99.998%	0.25%	99.990%	0.43%	99.983%
	Sp 0.99	Se0.34	0.36%	99.993%	18.01%	99.571%	0.13%	99.997%	0.74%	99.985%	1.26%	99.975%
	Se0.95	Sp 0.5	0.02%	99.999%	1.21%	99.935%	0.01%	100.000%	0.04%	99.998%	0.07%	99.996%
	Se0.99	Sp 0.14	0.01%	99.999%	0.74%	99.954%	0.00%	100.000%	0.03%	99.998%	0.04%	99.997%
	Youden		0.04%	99.998%	2.52%	99.869%	0.02%	99.999%	0.09%	99.996%	0.15%	99.992%
BARD1-CA125 CV validation set average	Sp 0.95	Se0.86	0.18%	99.998%	10.00%	99.905%	0.07%	99.999%	0.37%	99.997%	0.64%	99.994%
	Sp 0.99	Se0.74	0.79%	99.997%	32.35%	99.831%	0.28%	99.999%	1.59%	99.994%	2.70%	99.990%
	Se0.95	Sp 0.78	0.05%	99.999%	2.71%	99.959%	0.02%	100.000%	0.09%	99.999%	0.16%	99.998%
	Se0.99	Sp 0.25	0.01%	100.000%	0.85%	99.974%	0.01%	100.000%	0.03%	99.999%	0.05%	99.998%
	Youden		0.13%	99.999%	7.51%	99.917%	0.05%	100.000%	0.27%	99.997%	0.47%	99.995%

PPV, positive predictive value; NPV, negative predictive value; Se, sensitivity; Sp, specificity; BRCAm, BRCA1/2 mutation carriers; <50, 50–64, >50, women less than 50, between 50–64, or more than 50 years of age.

## Supplementary Materials

The following are available online at https://www.mdpi.com/article/10.3390/genes12070969/s1, Table S1: GUM 261 annotation.
